# Knowledge of breast cancer and its early detection measures among rural women in Akinyele Local Government Area, Ibadan, Nigeria

**DOI:** 10.1186/1471-2407-6-271

**Published:** 2006-11-26

**Authors:** O Abimbola Oluwatosin, Oladimeji Oladepo

**Affiliations:** 1 Department of Nursing, Faculty of Clinical Sciences, College of Medicine, University of Ibadan, Oyo state, Nigeria; 2 Department of Health Education and Health Promotion, Faculty of Public health, College of Medicine, University of Ibadan, Oyo state, Nigeria

## Abstract

**Background:**

Breast cancer is the commonest cancer among women in Nigeria and globally. In Nigeria, late presentations of breast cancer cases have also been consistent for three decades. In an environment where there is no established national screening program for breast cancer, it is pertinent to assess the knowledge of breast cancer and its early detection measures. The objective of this study therefore, was to assess rural women's level of knowledge of breast cancer and its early detection measures.

**Methods:**

The knowledge of various aspects of breast cancer; etiology, early warning signs, treatment modes and early detection measures; was assessed among women in two randomly selected health districts in Akinyele Local Government in Ibadan. The assessment was performed with the use of a self-structured validated questionnaire administered by trained interviewers to 420 women randomly selected from the two health districts. The various aspects of facts about breast cancer were scored and added together to determine respondents' level of knowledge

**Results:**

The mean score of knowledge of breast cancer was 55.4 SD 5.4 (range of scores obtainable was 26–78), while the mean score for knowledge of early detection of breast cancer was 24.8 SD 2.3 (range of scores obtainable was 12–36). The leading source of information about breast cancer was "elders, neighbors and friends" and 63(15.4%) acknowledged this source, while only 18 (4.4%) respondents acknowledged health workers as source. Only 54 (13.3%) claimed to have heard about breast self- examination (BSE) however, and the leading source of information about BSE were health workers. Nine (2.2%) of respondents claimed this source.

**Conclusion:**

This study revealed that respondents lacked knowledge of vital issues about breast cancer and early detection measures. It also revealed that health workers were not forthcoming with information to the public thereby constituting a challenge to community health nurses and other health workers, to provide vital information to the public.

## Background

Breast cancer is the commonest cancer among women in the world and in Nigeria [1]. In Nigeria the prevalence of breast cancer is 116 per 100,000 and 27,840 new cases were expected to develop in 1999[1]. Recent observations show that the frequency of breast cancer has risen over that of non-Hodgkin's lymphomas and cervical cancer in Nigeria[2]. This trend was attributed to several factors; the acceptance of fine needle aspiration as an accurate diagnostic evaluation, increased awareness about breast cancer and usefulness of breast self-examination [2].

The relative frequencies of breast cancer among other female cancers, from Cancer Registries in Nigeria were 35.3% in Ibadan, 28.2% in Ife-Ijesha, 44.5% in Enugu, 17% in Eruwa, 37.5% in Lagos, 20.5% in Zaria and 29.8% in Calabar [3]. In all the centers, except Calabar and Eruwa, breast cancer rated first among other cancers. Further reports showed that majority of cases occurred in pre menopausal women, and the mean age of occurrence ranged between 43–50 years across the regions. The youngest age recorded was 16 years, from Lagos [3]. Adebamowo and Ajayi [1] also reported that peak age of incidence in Nigeria is 42.6 years, and that 12% of cases occurred before 30 years while postmenopausal women accounted for 20% of cases. These authors were of the opinion however that "...these parameters may be more reflective of the demographic profile of Nigeria than an inherent difference in epidemiological characteristics of breast cancer in Nigeria..." [1].

The predominant feature of late presentation of breast cancer had been reported over three decades in Nigeria [4-9]. This is probably due to the fact that there is no established national screening program for breast cancer. Awareness of early detection measures of breast cancer such as clinical breast examination (CBE) and breast self examination (BSE) is also low [1]. In an environment where late presentation is predominant and where most breast cancers were detected accidentally by women themselves [1] there is an urgent need for awareness of breast cancer and its early detection measures. Similarly, baseline reports on current level of knowledge would be vital to an effective awareness program, hence the need for studies assessing level of knowledge of breast cancer in the population [1]. The diagnosis of breast cancer is a topic that is not freely discussed, hence lack of knowledge prevails. It has also been documented that rural population is usually neglected in health education issues [10] hence the selected population. A recent study reported that 73 out of 326 breast cancer patients recruited for study initially were women who lived predominantly in the rural area [11].

The women in the districts studied, have access to a primary health care center (PHC) which only deals with minor ailments and treatment of common diseases. Women make use of the health facility basically for maternity and child care. Since there is no established National screening program for breast cancer, this study may provide evidence for the requirement of such in the future and also provide justification for training of Nurses in PHC to perform clinical breast examination and teach clients breast self examination.

## Objective of study

To assess rural women's level of knowledge of breast cancer and its early detection measures.

## Methods

A Multistage sampling technique, that included the following variables: health district, villages; households and the unit of enquiry, was utilized. Two health districts, Alabata and Iroko, were randomly selected among the 10 health districts that met the criteria for accessibility and being rural, in Akinyele Local Government Area. In order to have a sample that would be representative of the health districts, villages in each health district were categorized into three based on distance to the primary health care center. Category A was the village, which housed the PHC, B villages were those within 5 km radius to the PHC, and C villages were those > 5 km walking distance to the PHC.

Two villages each were randomly selected from group B and three villages each from group C. In each village the acknowledged center of the village was located and the central house was selected subsequently. Every third house on either side was selected. One woman was selected in each household based on the willingness to participate.

The sample size was calculated using the projected female population in the two health districts (4135), and the following parameters: worst accepted value at 0.2%, frequency expectancy, 2.2%, based on previous report of BSE practice finding [13] which acknowledged that only 2.2% of women carried out BSE, at 95% confidence interval. Based on these parameters the calculated sample size came to 375. However 420 (representing 10% of 41135 (the projected female population calculated from the 1991 population census) women were interviewed. The inclusion criteria were that a woman must be aged 20–60 years, and must have been resident in the village for a minimum of six months.

The instrument used for data collection was a structured questionnaire developed by the author and modified based on findings of focus group discussions. The questionnaire was translated to the local language, Yoruba. The test, retest reliability of the instrument was conducted using 20 women, and their data was not part of the sample. The reliability was calculated using correlation coefficient and r *= 0.95*. As this report was part of a larger study, the questionnaire consisted of four sections, with a total of 56 items. Questions on demography, knowledge of incidence, cause and risk factors of breast cancer, knowledge of early warning signs of breast cancer, knowledge of early detection measures were considered for the purpose of this paper.

Interviews were conducted by research assistants specifically trained for this study and one of the researchers. The research assistants were also experienced in data collection for community studies. The interview was conducted on one to one basis, that is just the interviewer and the woman but it was within the reach of others. This was based on the culture in the rural communities. Some women declined participating in the study but data collection continued until 420 interviews were carried out. Daily post field verbal reports during data collection showed that most of the women who declined were the elderly. A written record was not kept.

Data analysis was carried out using EPI INFO statistical package and descriptive statistics of frequency and percentages were used. The knowledge score was calculated by assigning 3 to the correct response, 1 to the wrong response, and 2 to "I don't know". Scores from each of the following aspects: knowledge of risk factors, knowledge of early warning signs and knowledge of treatment of breast cancer were pulled together to have the overall knowledge score. The expected minimum score was 26 and the maximum 78.

### Ethical consideration

The chairman of the Local government gave official permission to use the setting for the research. Through the chiefs of the selected villages a meeting was organized with women and women leaders. At that meeting, adequate information was given about the study. After this, a meeting with women in the community was held and there, thorough explanation of the study was given emphasizing their right to decide to participate or not. The researcher enjoyed maximum cooperation in the community.

## Results

Four hundred and seven (96.6%) out of 420 questionnaires were found adequate for analysis. The mean age of respondents was 37.4 (S.D. 12.5) years. Those that were married among them constituted 82.9%. They were predominantly Muslims, 55.6%, while 43.8% were Christians. 56.5% had no formal education, and only 26.1% had primary school education (see table 1)

### Knowledge of cause/risk factors for breast cancer

Only 212 (52%) identified that the cause of breast cancer is unknown. Table 2 shows respondents' knowledge of risk factors for breast cancer.

### Knowledge of early warning signs of breast cancer

In considering respondents' knowledge of early warning signs of breast cancer, 300 (73.7%) of the respondents claimed that they did not know any warning signs. Only eight, (1.9 %) identified that a painless lump could be a warning sign of breast cancer. Twenty six (6.4%) acknowledged swelling, only one person (0.2%) acknowledged breast skin changes, two (0.5%) acknowledged discharge from the nipples and another four (1%) considered nipple retraction as a warning signs of breast cancer. Other signs identified by one respondent each were fever, purities, cold, weight loss and presence of a wound. Six respondents (1.5%) identified pain as an early warning sign of breast cancer.

### Knowledge of "treatment of breast cancer"

Three hundred and forty three (90.7%) of the respondents did not know anything about treatment of breast cancer. Eighteen (4.8%) identified use of drugs (hormone replacement/chemotherapy), two (0.5%) acknowledged surgery and one respondent identified the combination of chemotherapy, surgery and radiotherapy. More than half of the respondents 224 (55.2%) however agreed that early treatment of breast cancer might prevent death while 15 (3.7%) did not support that early treatment may prevent death. One hundred and sixty seven (41%) claimed that they did not know.

Each of the following aspects: knowledge of risk factors, knowledge of early warning signs and knowledge of treatment of breast cancer were scored and the score pulled together. The overall mean knowledge score was 55.4 SD 5.4. The range of the scores was 34 – 70.

There was no significant difference in the mean knowledge scores across age groups p = 0.2. However the lowest mean knowledge score of 54.4 SD 4.8 was recorded among the 51– 60 years age group while the highest mean knowledge score of 55.9 SD 5.9 was recorded among the 41– 50 years age group. There was no significant difference in the knowledge mean scores based on marital status p = 0.3; but the married group had the highest mean score of 55.6 SD 5.6. There was also no significant difference in the mean knowledge score across educational groups p = 0.2.

### Knowledge of early detection measures of breast cancer

The respondents identified various measures that are not within the conventional methods of early detection measures. These include breast cleanliness, washing the nipples regularly, and traditional care among others. However only 26 (6.4%) identified BSE while only 5 (1.2%) identified clinical breast examination and none identified mammography as an early detection measures. Only 58 (14.3%) of the respondents knew that BSE should be performed 2–3 days after menstruation monthly and 43 (10.6%) knew that women who have reached menopause were expected to choose a specific day of the month to perform BSE.

Only 49 (12%) were aware of the three processes involved in BSE, that is, standing in front of a mirror to examine the breasts, lying down and while bathing. Twenty two (5.4%) acknowledged that women who are thin have the advantage of detecting breast lump easily while more, 33 (8.1%) agreed that it is more difficult for fat women to detect breast lump. Only 53 (13%) agreed that younger women (< 50 years) discover breast lump than the older women. The overall mean score was 24.8 SD 2.3 out of a minimum score of 16 and a maximum of 36.

### Practice of early detection measures

Three hundred and ninety four responded to the question "Have you ever examined your breast for early detection of breast cancer?" Three hundred and fifty one (89.1%) said NO, while only 43(10.9%) said "YES". However only 26 (6.4%) claimed to have examined their breasts by themselves, eight (2%) claimed to have been examined by health workers, two (0.5%) by their mothers, three (0.7%) by their mothers in law, another three (0.7%) by their husbands, and one (0.2%) was examined by a friend.

In response to the question: "how many times in a month do you perform breast self examination?" majority of the respondents, 323 (79.4%) acknowledged that they did not practice BSE. None examined their breasts once a month. One participant (0.2%), claimed to examine her breast six times a month. Another five (1.2%) acknowledged examining their breasts eight times a month, while 66 (16.2%) could not remember how many times a month they examined their breast.

### Source of information

Three hundred and forty nine (85.7%) of the respondents claimed to have heard about breast cancer but only 54 (13.3%) claimed to have heard about BSE. Respondents' leading source of information about breast cancer was "elders, neighbors and friends", sixty three (15.4%) acknowledged this source. Twenty two (5.4%) acknowledged television and radio, 21(5.2%) acknowledged getting information from those that had the disease while only 18 (4.4%) acknowledged health workers as their source of information. However the leading source of information about BSE was health workers, nine (2.2%) of respondents claimed this source while two (0.5%), acknowledged television/radio and one (0.2%) claimed "elders neighbors and friends" and another acknowledged the questionnaire as the first source of information.

## Discussion

Formal education provides an advantage in the understanding of various health issues. This study revealed that majority of the population had no formal education. The educational structure is different from that of a study conducted in Port Harcourt (a major city in Nigeria) in which 98% of the study population had formal education [12]. Another study [13] in a sub urban population also reported that 33.2% of the population had no formal education. These findings affirm the educational disparity between rural and city dwellers. More than half of the study population was in the 20–40 year age group. This reflects the experience on the field whereby many of the older women declined participating in the study, giving the reason that they were past childbearing.

The knowledge of risk factors for breast cancer in this study was poor. This finding is similar to that of previous studies [12,14]. Majority of the respondents were not aware of early warning signs of breast cancer. Only 1.9% acknowledged a painless lump as an early warning sign. This is similar to a previous report from Ibadan about a decade ago [7] but contrary to the finding of a study [14] among teachers. The lack of knowledge may be explained by the findings that the leading source of information about breast cancer was "elders, neighbors and friends". One can therefore imply that lack of knowledge of accurate information about breast cancer exists in the community. There is therefore a need to emphasize during breast cancer awareness programs, that a painless lump is significant, though pain is considered a general indicator for impending danger. The finding that six respondents considered that breast wound is an early warning sign of breast cancer reiterates the need for specific emphasis on early warning signs during beast cancer awareness in the community.

The overall knowledge of treatment of breast cancer is also very poor perhaps because typically in Nigeria especially among the low socioeconomic class, patients and relations usually do not bother about the details of treatment. The treatment is usually left to the discretion of the health care provider. This was also observed in a previous study among Egyptians [15]. It is disturbing that few of the participants (3.7%) did not think that early treatment may prevent death. This may be an indication that the myth "that breast cancer means death" is rooted in this community.

One of the local names for breast cancer in the setting for this study is "jejere". "Jejere" means "that which devours". This among others has implications for community health nurses' breast cancer awareness programs.

The finding that none of the respondents acknowledged mammography as an early detection measure is expected since mammography is not readily available to this population [1]. The findings that few of the participants acknowledged BSE as an early detection measure, and that the very few that practiced it did not do it according to the classic BSE technique is of a major concern in an environment where this seems to be the only easily accessible early detection measure for breast cancer. On the contrary, previous studies in sub urban and urban communities in Nigeria [12,13] have reported higher percentages of population practicing BSE. However, they did not practice BSE according to the classic technique. A previous study [16], in a rural community in India reported that none of the participants had ever practiced BSE.

Findings also showed that the recommended clinical examination once a year is not popular. Various groups – mothers, mother in-laws, husbands, and friends were reported to conduct breast examination for the participants. In an environment where there is no active national screening programme [1], this is of great concern.

More respondents were better informed about breast cancer than about BSE. The leading source of information on breast cancer was "elders, neighbors and friends", whereas a previous study in Northern Nigeria [6] had reported electronic media as leading source of information on breast cancer. For BSE on the other hand, the leading source of information was health care workers. This finding is similar to those of previous studies [12,13]. It is understandable that health care workers have consistently been reported as the leading source of information for BSE.

## Conclusion

The findings of this study have further provided evidence that rural women lack appropriate information about breast cancer and its' early detection measures. The finding that the major source of information about beast cancer was "elders, neighbors and friends" suggests that health care workers are yet to succeed in their role of providing health information. This role needs to be revised and it is hoped that the findings will help in the preparation of breast cancer specific health education.

## Limitation

The authors acknowledged the study limitations of no record of number and characteristics of people that refused to participate in the study.

## Competing interests

The author(s) declare that they have no competing interests.

## Authors' contributions

AO initiated the study. AO developed the instrument, conducted and supervised data collection, conducted data analysis and wrote the manuscript.

OO supervised every stage of the study and provided valuable suggestions for the design of the study. He read the draft of the manuscripts and made suggestions that were used to produce the final manuscript.

## Pre-publication history

The pre-publication history for this paper can be accessed here:



## Figures and Tables

**Table 1:** Demographic characteristics of respondents.

**Demographic characteristics**	**Category**	**Frequency**	**Percentage**
Age group N = 405	20–30 years	165	40.7
	31–40 years	97	24
	41–50 years	80	19.8
	51–60 years	63	15.5

Marital status N = 403	Married	334	82.8
	Separated	8	2.0
	Widow	45	11.2
	Single	14	3.5
	Divorced	2	0.5

Religion N = 390	Traditional	2	0.6
	Muslim	217	55.6
	Christian	171	43.8

Educational status N = 402	No formal education	227	56.5
	Informal education	7	1.7
	Primary school	105	26.1
	Junior secondary	32	8
	Senior secondary	24	6
	Tertiary	7	1.7

**Table 2:** Respondents' knowledge of risk factors.

**Risk factor**	**Frequency of positive response**	**Percentage of sample**
Fat women are more prone to breast cancer than slim women.	88	21.6
Taking of oral contraceptive pills is a risk for breast cancer.	71	17.4
Prolonged breastfeeding is a risk for developing breast cancer.	157	38.6
Early menarche and late menopause is a risk for developing breast cancer.	159	39.1
Delayed first pregnancy is a risk for developing breast cancer.	43	10.5
Infertility is a risk for developing breast cancer.	34	8.3
Breast cancer is more common among older women than younger ones	80	19.6
Multiparity is a known risk for breast cancer.	148	36.4
Prolong breast feeding is not a known risk for breast cancer	176	43.2
